# Supplementary material for: Defying Gravity to Enhance Power Output and Conversion Efficiency in a Vertically Oriented Four-Electrode Microfluidic Microbial Fuel Cell
Source: Micromachines (Basel). 2024 Jul 27;15(8):961. doi: 10.3390/mi15080961 (PMC11356121; doi:10.3390/mi15080961)
Supplement: Supplementary file 1 [file micromachines-15-00961-s001.zip › micromachines-3114245-supplementary.pdf]

Supporting information

Defying Gravity to Enhance Power Output and Conversion Efficiency in a Vertically Oriented Four-Electrode Microfluidic Microbial Fuel Cell

Linlin Liu [1], Haleh Baghernavehsi [1] and Jesse Greener [1,2,*]

[1] Département de Chimie, Université Laval, Québec, QC G1V 0A6, Canada

[2] CHU de Québec, Centre de Recherche du CHU de Québec, Université Laval, Québec, QC G1L 3L5, Canada

* Correspondence: jesse.greener@chm.ulaval.ca; Tel.: +418-656-2131

Supplementary Sections

S1. Power density measurements of young MFCs without precautions against gravitational effects

S2. Mature EAB power density and polarization curves

S3. Computational fluid dynamic and electrochemical simulation of electrode activity in a 4-electrode microfluidic MFC

S1. Power density measurements without precautions against gravitational effects

As explained in the main paper, electroactive biofilm (EAB) growth following inoculation was heavily impacted by the anode orientation. The bottom-side anodes (upward-facing) always grew faster and more efficiently than the top-side anode (downward-facing). We repeated the inoculation and initial growth process 5 times, always using the same procedure, including total flow rates of Q=0.6 mL h^{-1}. In Figure S1, we reproduce two of those results (power density and polarization curves) after an EAB growth time of approximately 20 days. Measurements included slight

modifications in the method and experimental conditions during data acquisition, such as using constant resistance technique at low total flow rate of Q=0.6 mL h^{-1} (Figure S1a) and linear scan voltammetry at a high total flow rate Q=6mL h^{-1} (Figure S1b). In both cases, the disparity in performance between upper (MFC_a) and lower

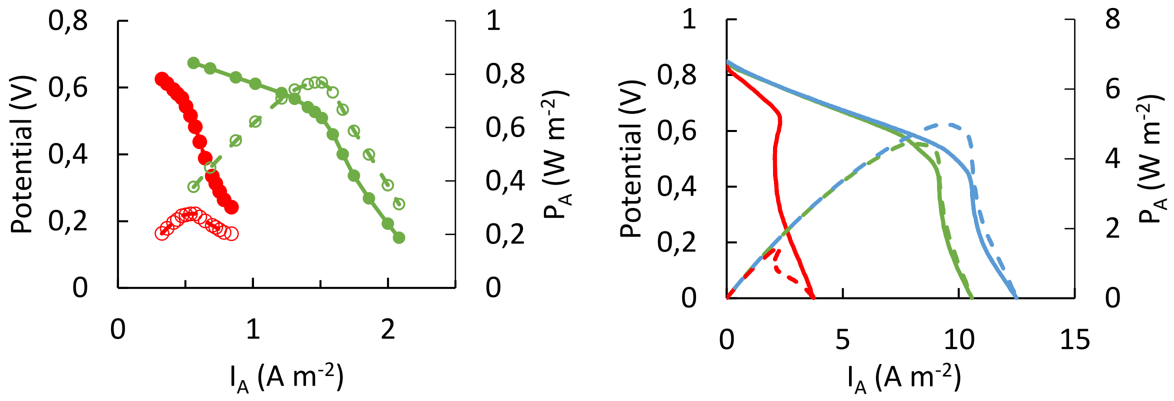

(MFC_b) electrode pairs was the same as shown in the main paper.

Figure S1. Polarization (solid) and power density (dash) curves from two different 4-electrode MFC with independent analysis of top electrode pair (MFC_a; red) and bottom electrode pair (MFC_b; green) after growth for approximately 20 days under low flow rate of Q_{Ac}=0.5 and Q_{Fe}=0.3 mL h^{-1}. Data was acquired by constant resistance method with a hold time of 1 hour (a) and by LSV with scan rate of 2 mV s^{-1} (b) at a high flow rate Q_{Ac}=4 and Q_{Fe}=2 mL h^{-1}. LSV analysis was also conducted on the 4-electrode MFC with a parallel connection between individual electrode pairs ($MFC_{a||b}$; blue).

S2. Mature EAB power density and polarization curves at low flow rate.

To complement the power density and polarization curves of the mature 4-electrode MFC at high flow rate shown in the main paper (Figure 4), we provide data showing the results from the same device at low flow rate (Figure S2). For all electrical configurations (including $MFC_{a||b}$) the power output of individual MFC_a and MFC_b remained normalized thanks to the inversion process described in the main paper, though raw power and current outputs are reduced compared to those acquired at high flow rate.

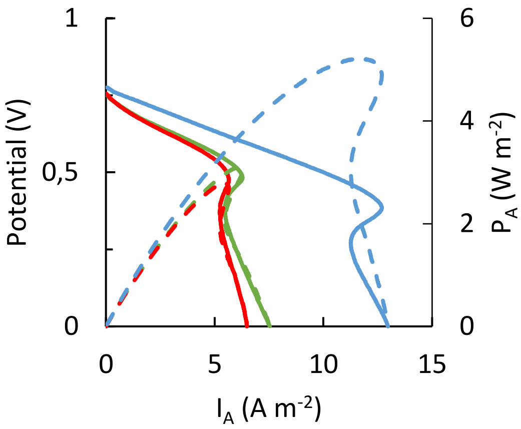

Figure S2. Power density (dashed) and polarization (solid) curves for a mature MFC after flipping at low flow rate (Q_{Ac}=0.4 mL h^{-1}, Q_{Fe}=0.2mL h^{-1}). Color coding includes: MFC_a (red), MFC_b (green), $MFC_{a||b}$ (blue). Data was acquired by linear scan voltammetry technique (2 mV s^{-1}).

S3. Computational fluid dynamic and electrochemical simulation of electrode activity in a 4-electrode microfluidic MFC

We present additional results for the computational fluid dynamic electrochemical simulation presented in the main paper. In Figure S3a we present the computed acetate concentration [Ac] across the microfluidic MFC device cross-section for the 2- and 4-electrode configuration. Starting from the anode side (0 mm) the profile shows a local reduction in [Ac] at the position closest to the cathode. But very limited reduction in concentration on the electrode far from the cathode. In the region where consumption was strongest, the figure shows that local consumption was approximately 5 times higher for the 4-electrode MFC compared to the 2-electrode version. The Figure S3a.

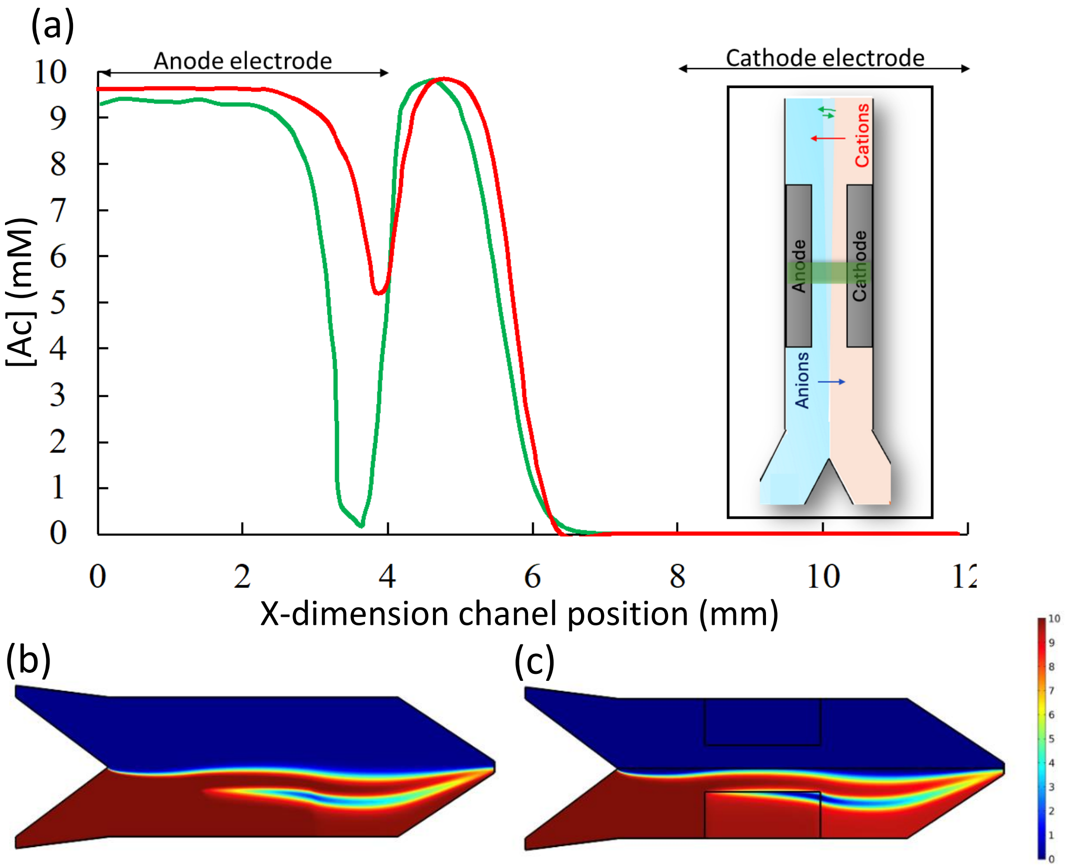

Figure S3. (a) Comparison of acetate consumption along different lateral positions within the microfluidic MFC for a 4-electrode MFC ($MFC_{a||b}$; green) and a 2-electrode MFC (i.e., MFC_a or MFC_b; red). Inset (black box) shows the position where the concentration profile was acquired (green path). Simulations of the acetate concentration for a 2-electrode MFC (b) and a 4-electrode MFC (c). Concentration color bar is in units of mM.
